# Supplementary material for: A comprehensive risk score for effective risk stratification and screening of nasopharyngeal carcinoma
Source: Nat Commun. 2021 Aug 31;12:5189. doi: 10.1038/s41467-021-25402-z (PMC8408241; doi:10.1038/s41467-021-25402-z)
Supplement: Supplementary file 1 — Supplementary Information [file 41467_2021_25402_MOESM1_ESM.pdf]

Title: A Comprehensive Risk Score for Effective Risk Stratification and Screening of Nasopharyngeal Carcinoma

Authors: Xiang Zhou[1], Su-Mei Cao[1], Yong-Lin Cai[2], Xiao Zhang[1], Shanshan Zhang[1], Guo-Fei Feng[3], Yufeng Chen[4], Qi-Sheng Feng[1], Yijun Chen[1], Ellen T. Chang[5,6], Zhonghua Liu[7], Hans-Olov Adami[4,8], Jianjun Liu[9,10], Weimin Ye[4,11"], Zhe Zhang[3]*, Yi-Xin Zeng[1]*, and Miao Xu[1]*

Affiliations:

[1] State Key Laboratory of Oncology in South China, Collaborative Innovation Center for Cancer Medicine, Guangdong Key Laboratory of Nasopharyngeal Carcinoma Diagnosis and Therapy, Sun Yat-sen University Cancer Center, Guangzhou, P. R. China

[2] Department of Clinical Laboratory, Wuzhou Red Cross Hospital, Wuzhou, China

[3] Department of Otolaryngology/Head and Neck Surgery, First Affiliated Hospital of Guangxi Medical University, Nanning, China

[4] Department of Medical Epidemiology and Biostatistics, Karolinska Institutet, Stockholm, Sweden

[5] Center for Health Sciences, Exponent, Menlo Park, CA, USA

[6] Stanford Cancer Institute, Stanford, CA, USA

[7] Department of Statistics and Actuarial Science, The University of Hong Kong, Hong Kong SAR, China

[8] Clinical Effectiveness Group, Institute of Health and Society, University of Oslo, Norway

[9] Human Genetics, Genome Institute of Singapore, Agency for Science, Technology and Research (A*STAR), Singapore

[10] Department of Medicine, Yong Loo Lin School of Medicine, National University of Singapore, Singapore

[11] Department of Epidemiology and Health Statistics & Key Laboratory of Ministry of Education for Gastrointestinal Cancer, Fujian Medical University, Fuzhou, China

These authors contributed equally: Xiang Zhou, Su-Mei Cao, Yong-Lin Cai and Xiao Zhang.

*Corresponding authors.

Corresponding authors:

Zhe Zhang, MD, PhD, Department of Otolaryngology/Head and Neck Surgery, First Affiliated Hospital of Guangxi Medical University, Nanning 530021, China; E-mail: zhangzhe@gxmu.edu.cn

Yi-Xin Zeng, MD, PhD, State Key Laboratory of Oncology in South China, Collaborative Innovation Center for Cancer Medicine, Guangdong Key Laboratory of Nasopharyngeal Carcinoma Diagnosis and Therapy, Sun Yat-sen University Cancer Center, Guangzhou 510060, China; E-mail: zengyx@sysucc.org.cn

Miao Xu, PhD, State Key Laboratory of Oncology in South China, Collaborative, Innovation Center for Cancer Medicine, Guangdong Key Laboratory of Nasopharyngeal Carcinoma Diagnosis and Therapy, Sun Yat-sen University Cancer Center, Guangzhou 510060, China; E-mail: xumiao@sysucc.org.cn

Supplementary Figure 1

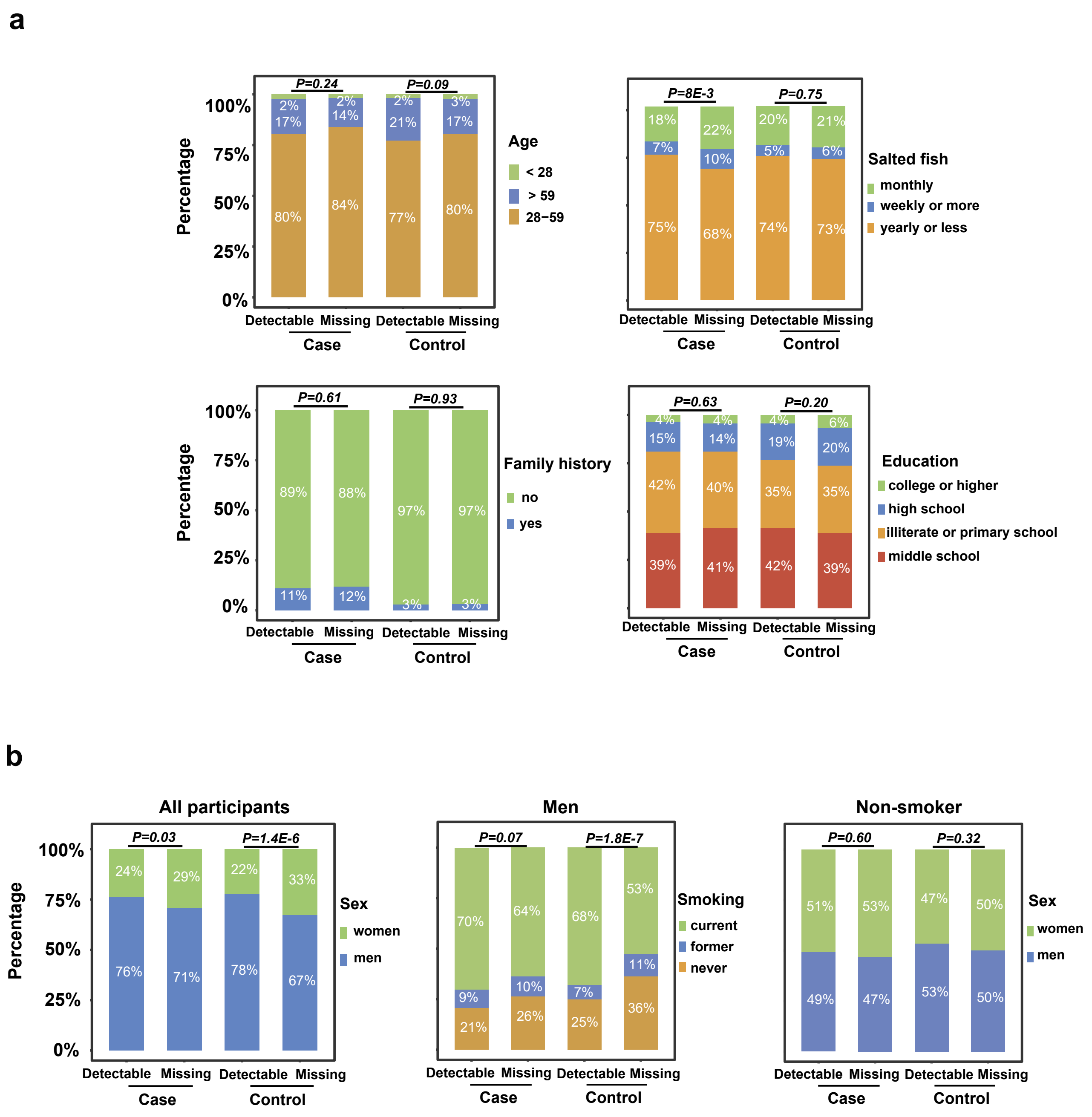

Supplementary Figure 1. Distribution of genotyping success or failure in all study participants. (**a**) Stacked bar plots indicate the distribution of genotyping success or failure for EBV SNPs (162215, 162476 and 163364). (Variables: age, education, salted-fish consumption and family history). (**b**) Stacked bar plot representation of the distribution of EBV genotyping success or failure by sex in all participants, by smoking status in men and by sex among non-smokers recruited in this study. The P values were calculated using χ^2 tests.Source data of (**a, b**) are provided in the Source Data file.

Supplementary Figure 2

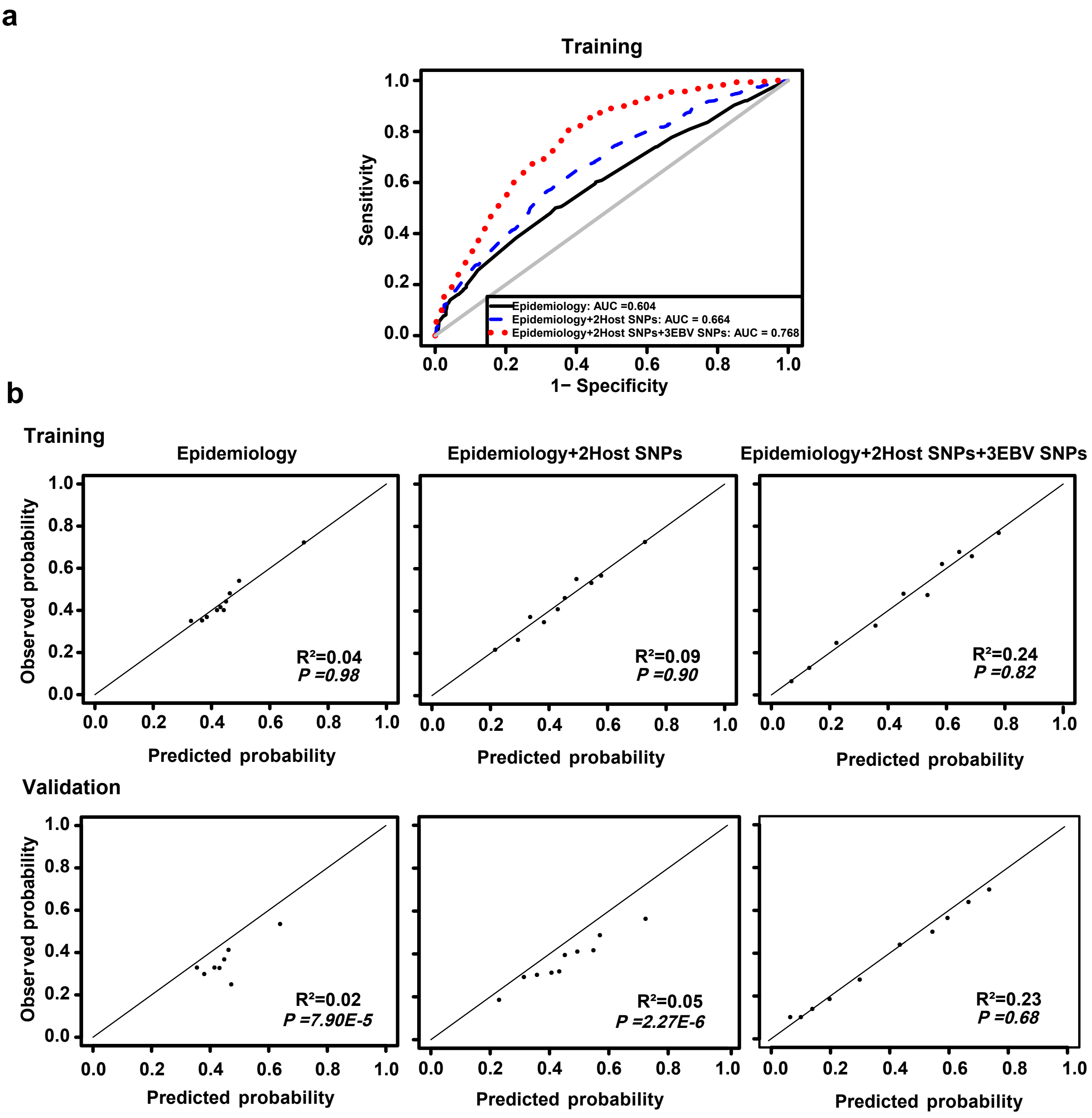

Supplementary Figure 2. Evaluation of comprehensive risk score (CRS) for NPC risk prediction. (**a**) Receiver operating characteristic (ROC) curve analysis of the training dataset. The area under the ROC curve (AUC) for each model is indicated. (**b**) Hosmer-Lemeshow goodness-of-fit analyses of the three risk prediction models in training dataset (upper panel) and validation dataset (lower panel). Blackspot: the observed NPC event rates (y-axis) *versus* the expected event rates (x-axis) as predicted by the three risk prediction models indicated for 10 groups of equal sample size in training dataset (upper panel) and validation dataset (lower panel). Diagonal: the line of perfect fit between the observed and predicted probability. R^2, Nagelkerke pseudo R-squared. *P* : Two-sided pearson's chi-squared goodness-of-fit test. The three risk prediction models: Epidemiology, model #1; Epidemiology + 2 Host SNPs, model #2; Epidemiology + 2 Host SNPs + 3 EBV SNPs, model #3 (the CRS model).

Supplementary Figure 3

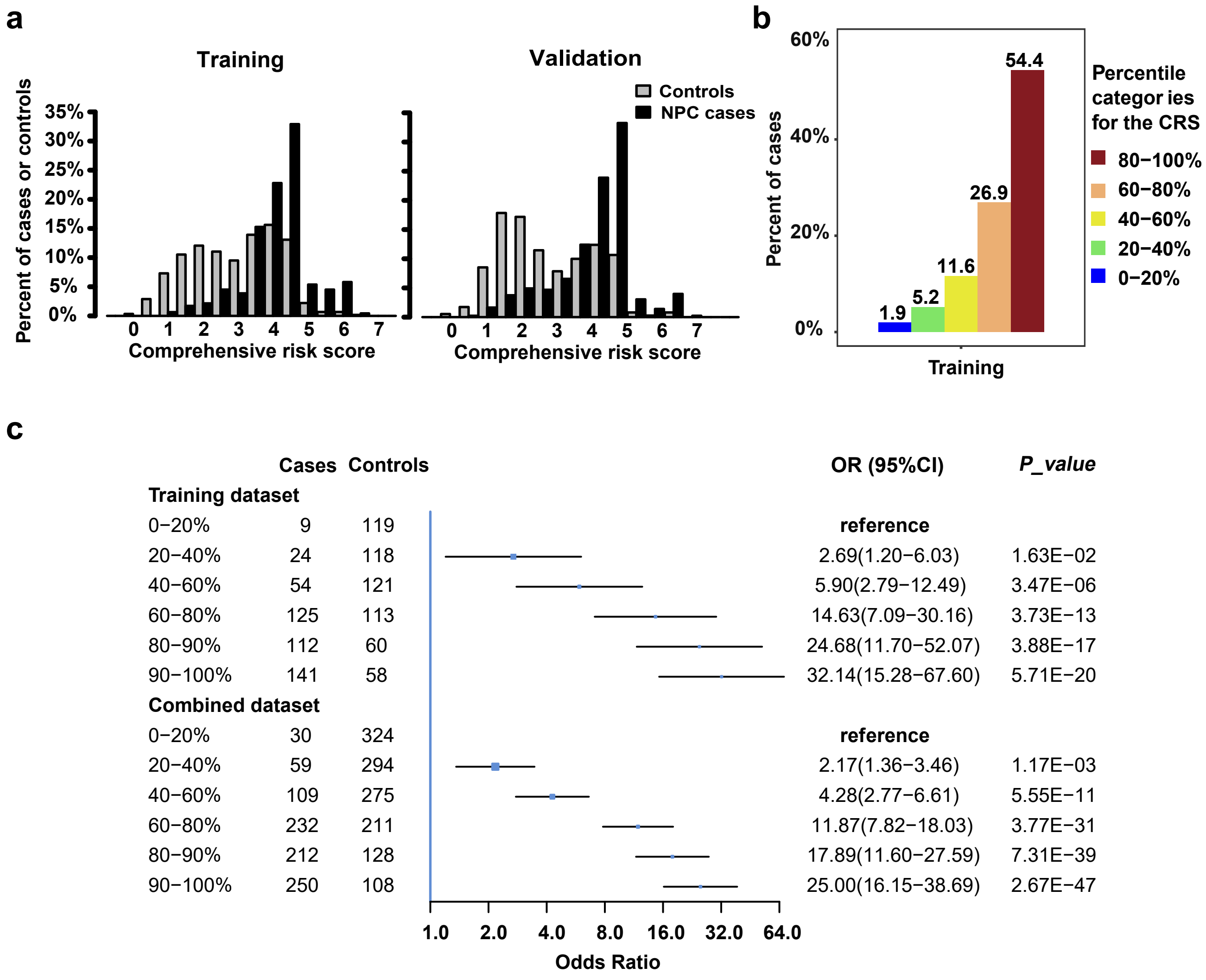

Supplementary Figure 3. NPC risk stratified by comprehensive risk score (CRS). (**a**) Distribution of the comprehensive risk score (CRS) among patients with NPC and controls in the training and validation datasets. (**b**) Distribution of patients stratified by the percentile of the CRS among controls. Study subjects in the training dataset were stratified into five categories according to the CRS percentile (0-20%, 20-40%, 40-60%, 60-80% and 80-100%) among the controls in the training dataset. (**c**) Associations between the CRS and NPC risk in the training (Cases: n=465, Controls: n=589) and combined datasets (Cases: n=892, Controls: n=1340). The training and combined datasets were stratified into five categories (lower four quintiles and top two deciles) according to the CRS percentile among the controls in the training dataset; participants in the bottom quintile of CRS served as the reference group. The odds ratio (OR) of developing NPC was estimated using logistic regression analysis with each group included as a categorical variable. The blue squares represent the odds ratios of each category, and the error bars represent the 95% confidence intervals. CI, confidence interval. Source data of (**a**,**b**) are provided in the Source Data file.

Supplementary Figure 4

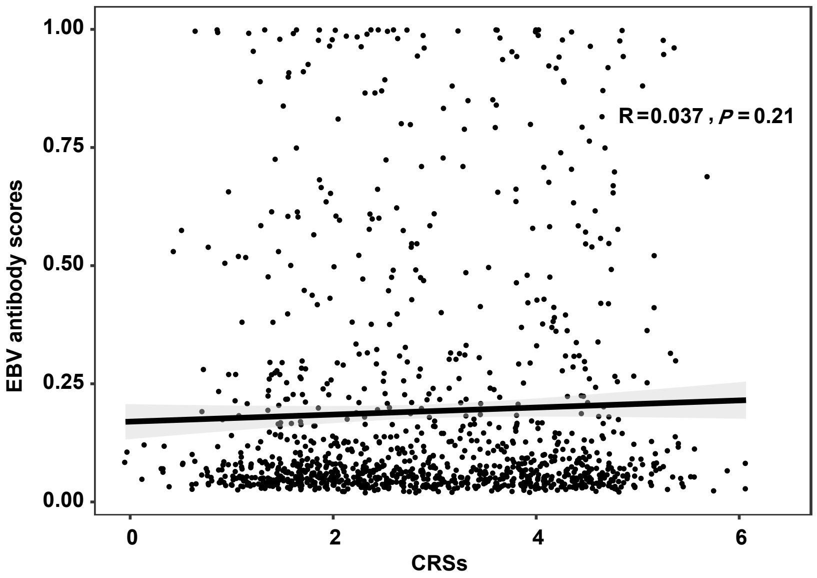

Supplementary Figure 4. Correlation between EBV antibody scores and CRS among the healthy controls in this study. The straight black line represents the best linear fit to our data, and the gray band represents a 95% confidence level. R: Pearson's correlation coefficient. The *P* value was calculated using a two-sided paired t-test.

Supplementary Figure 5

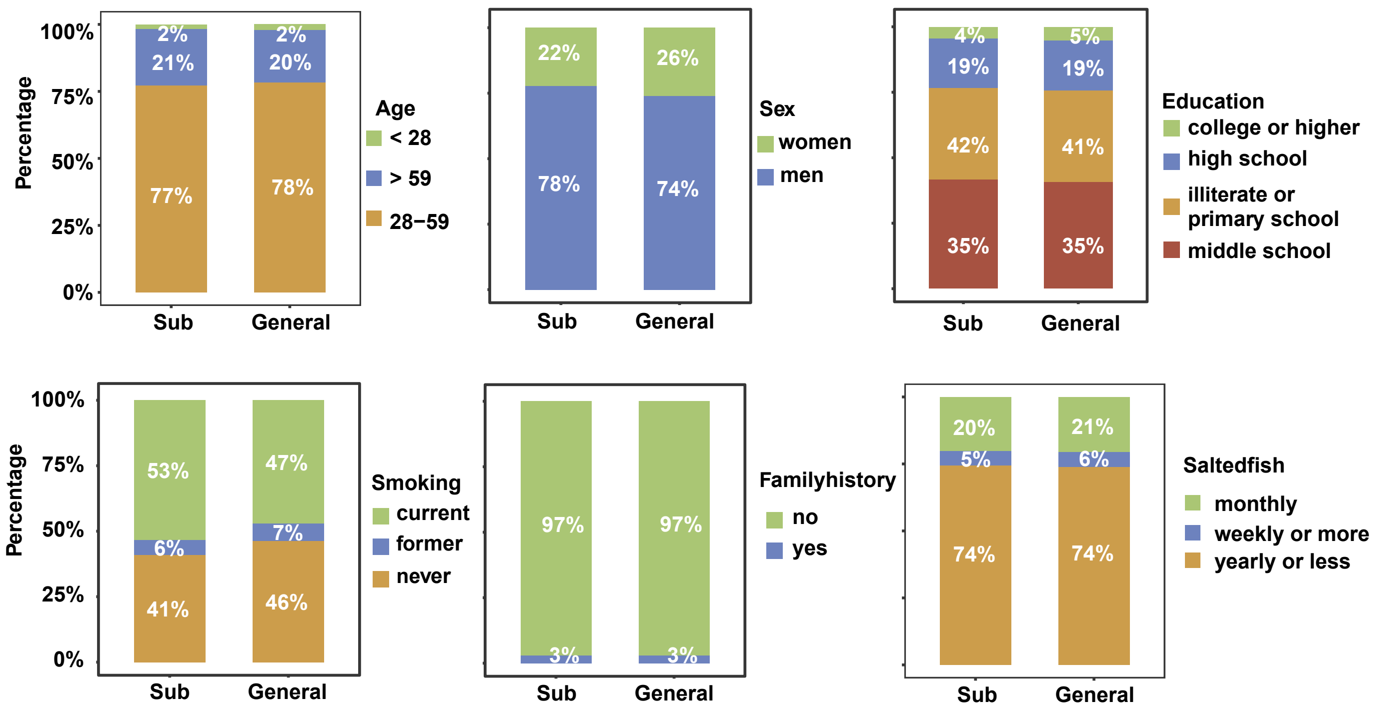

Supplementary Figure 5. Stacked bar plots indicate the distribution of epidemiological risk factors (age, sex, education, salted-fish consumption, smoking, and family history) in the analytic controls and in the population controls of the complete case-control study. Source data are provided in the Source Data file.

Supplementary Tables

Supplementary Table 1. Basic characteristics of the patients with NPC and the controls stratified by host risk alleles and EBV risk genotype.

Training dataset	Gene region	Cases (465)		Controls (589)		OR (95% CI)	*P* value*
		Low-risk Allele	High-risk Allele	Low-risk Allele	High-risk Allele		
**Host risk allele**							
rs2860580_C	*HLA-A*	228 (24.5)	702 (75.5)	433 (36.8)	745 (63.2)	1.79 (1.47 - 2.18)	6.18E-09
rs2894207_T	*HLA-B/C*	124 (13.3)	806 (86.7)	257 (21.8)	921 (78.2)	1.86 (1.46 - 2.36)	4.53E-07
rs1412829_T	*CDKN2A/2B*	91 (9.8)	839 (90.2)	150 (12.7)	1028 (87.3)	1.35 (1.01 - 1.79)	4.09E-02
rs9510787_G	*TNFRSF19*	544 (58.5)	386 (41.5)	746 (63.3)	432 (36.7)	1.23 (1.03 - 1.47)	2.24E-02
rs28421666_A	*HLA-DQ/DR*	69 (7.4)	861 (92.6)	108 (9.2)	1070 (90.8)	1.25 (0.92 - 1.70)	1.53E-01
rs31489_C	*CLPTM1L*	194 (20.9)	736 (79.1)	278 (23.6)	900 (76.4)	1.17 (0.95 - 1.44)	1.41E-01
rs6774494_A	*MDS1-EVI1*	292 (31.4)	638 (68.6)	408 (34.6)	770 (65.4)	1.16 (0.96 - 1.40)	1.14E-01
**EBV risk genotype**							
EBV162215_C	*BALF2-V700L*	19 (4.1)	446 (95.9)	157 (26.7)	432 (73.3)	8.63 (5.25 - 14.20)	2.08E-17
EBV162476_C	*BALF2-I613V*	26 (5.6)	439 (94.4)	206 (35.0)	383 (65.5)	9.06 (5.88 - 13.95)	1.67E-23
EBV163364_T	*BALF2-V317M*	70 (15.1)	395 (84.9)	317 (53.8)	272 (46.2)	6.63 (4.89 - 8.98)	4.45E-34

Validation dataset	Gene region	Cases (427)		Controls (751)		OR (95% CI)	*P* value*
		Low-risk Allele	High-risk Allele	Low-risk Allele	High-risk Allele		
**Host risk allele**							
rs2860580_C	*HLA-A*	213 (24.9)	641 (75.1)	502 (33.4)	1000 (66.6)	1.58 (1.30 - 1.92)	3.69E-06
rs2894207_T	*HLA-B/C*	107 (12.5)	747 (87.5)	272 (18.1)	1230 (81.9)	1.55 (1.21 – 1.98)	4.33E-04
rs1412829_T	*CDKN2A/2B*	77 (9.0)	777 (91.0)	139 (9.3)	1363 (90.7)	1.03 (0.77 - 1.38)	8.31E-01
rs9510787_G	*TNFRSF19*	497 (58.2)	357 (41.8)	941 (62.6)	561 (37.4)	1.19 (0.99 - 1.42)	6.08E-02
rs28421666_A	*HLA-DQ/DR*	57 (6.7)	797 (93.3)	137 (9.1)	1365 (90.9)	1.33 (1.00 - 1.78)	5.34E-02
rs31489_C	*CLPTM1L*	206 (24.1)	648 (75.9)	415 (27.6)	1087 (72.4)	1.21 (0.99 - 1.48)	5.66E-02
rs6774494_A	*MDS1-EVI1*	257 (30.1)	597 (69.9)	454 (30.2)	1048 (69.8)	0.99 (0.82 - 1.19)	8.79E-01
**EBV risk genotype**							
EBV162215_C	*BALF2-V700L*	28 (6.6)	399 (93.4)	242 (32.2)	509 (67.8)	6.75 (4.45 - 10.22)	2.23E-19
EBV162476_C	*BALF2-I613V*	59 (13.8)	368 (86.2)	397 (52.9)	354 (47.1)	7.13 (5.21 - 9.76)	1.64E-34
EBV163364_T	*BALF2-V317M*	104 (24.4)	323 (75.6)	513 (68.3)	238 (31.7)	6.84 (5.20 – 9.00)	5.88E-43

**P values* for host risk alleles and EBV risk genotype in association with NPC risk were calculated by logistic regression adjusted for age, sex, smoking, salted fish consumption and education.

Abbreviations: OR, odds ratio; CI, confidence interval.

Supplementary Table 2. Performance of the models for distinguishing NPC cases from population controls in training dataset.

Model	Training dataset					
	AUC	95%CI	Repeated 10-fold	*P value*‖	*P value*‖	R^2¶
Epidemiology model *	0.604	0.570-0.639	0.585	1.42E-18	------§	0.028
Epidemiology + 2Host SNPs model†	0.664	0.632-0.697	0.647	3.92E-14	3.29E-04	0.060
Epidemiology + 2Host SNPs + 3 EBV SNPs model‡	0.768	0.740-0.796	0.759	------§	1.42E-18	0.172

Abbreviations: AUC, area under the curve; CI, confidence interval; Repeated 10-fold, average AUC from repeated 10-fold cross validation.

* Epidemiological model: smoking, salted fish consumption, education and family history of NPC

† 2 Host SNPs: rs2860580, rs2894207

‡ 3 EBV SNPs: EBV162215, EBV162476 and EBV163364

§ "------" is a reference model

‖ The *P* value was calculated using a two-sided Delong's test.

¶ McFadden's Pseudo R^2

Supplementary Table 3. The performance of the models for distinguishing the patients with NPC from the controls in training and validation datasets.

Model	Training dataset					Validation dataset				
	AUC	95%CI	Repeated 10-fold	*P value*‖	R^2¶	AUC	95%CI	Repeated 10-fold	*P value*‖	R^2¶
3EBV SNPs model *	0.708	0.683-0.734	0.709	------§	0.132	0.745	0.719-0.771	0.743	------§	0.157
3EBV SNPs model + 2Host SNPs model†	0.755	0.727-0.784	0.750	1.51E-07	0.158	0.761	0.734-0.789	0.757	1.20E-02	0.163
3EBV SNPs model + Epidemiology model‡	0.742	0.713-0.771	0.727	1.92E-04	0.148	0.764	0.736-0.792	0.761	2.87E-03	0.170
3EBV SNPs model + 2Host SNPs + Epidemiology model	0.768	0.740-0.796	0.759	3.17E-10	0.172	0.772	0.745-0.800	0.770	1.74E-04	0.174

Abbreviations: AUC, area under the curve; CI, confidence interval; Repeated 10-fold, average AUC from repeated 10-fold cross validation.

*3 EBV SNPs: EBV162215, EBV162476 and EBV163364

†2 Host SNPs: rs2860580, rs2894207

‡ The epidemiological model included smoking, salted fish consumption, education and family history of NPC

§ "------" is a reference model

‖ The *P* value was calculated using a two-sided Delong's test.

¶ McFadden's Pseudo R^2

Supplementary Table 4. Net reclassification index for the risk prediction models.

Base model	NRI improvement (95%CI)		
Training	3EBV SNPs + 2Host SNPs model†	3EBV SNPs + Epidemiology model‡	3EBV SNPs + 2Host SNPs + Epidemiology model
3EBV SNPs model *	0.257(0.195-0.320)	0.143(0.098-0.188)	0.360(0.291-0.429)
3EBV SNPs model + 2Host SNPs model†	------	0.088(0.012-0.164)	0.149(0.099-0.199)
3EBV SNPs model + Epidemiology model‡		------	0.214(0.143-0.285)
3EBV SNPs model + 2Host SNPs + Epidemiology model			------
Validation			
3EBV SNPs model *	0.212(0.151 - 0.274)	0.086(0.050-0.121)	0.271(0.207-0.334)
3EBV SNPs model + 2Host SNPs model†	------	0.110(0.039-0.181)	0.102(0.062- 0.141)
3EBV SNPs model + Epidemiology model‡		------	0.190(0.125-0.254)
3EBV SNPs model + 2Host SNPs + Epidemiology model			------

Abbreviations: NRI, net reclassification index; CI, confidence interval.

Note: Reclassification was calculated for strata of predicted risks of < 0.2, 0.2 to 0.4, 0.4 to 0.6, 0.6 to 0.8 and > 0.8.

*3 EBV SNPs: EBV162215, EBV162476 and EBV163364

†2 Host SNPs: rs2860580, rs2894207

‡ The epidemiological model included smoking, salted fish consumption, education and family history of NPC

Supplementary Table 5. Prediction accuracy of the serum VCA-/EBNA1-IgA antibody tests and the models combining the serum EBV antibody test results and the comprehensive risk score.

Model	Cut off	Training dataset				Validation dataset			
		Sensitivity	Specificity	PPV	NPV	Sensitivity	Specificity	PPV	NPV
VCA-/EBNA1-IgA	P > =0.98	86.28%	96.03%	3.38%	99.98%	73.61%	98.36%	6.72%	99.96%
CRS (Top 40 percentile) + VCA-/EBNA1-IgA	CRS >=3.23 and P >=0.98	70.57%	98.96%	9.80%	99.95%	54.88%	99.85%	37.13%	99.93%
CRS (Top 30 percentile) + VCA-/EBNA1-IgA	CRS >=3.62 and P >=0.98	60.10%	99.58%	18.78%	99.94%	45.91%	100.00%	100.00%	99.91%
CRS (Top 20 percentile) + VCA-/EBNA1-IgA	CRS >=3.88 and P >=0.98	48.13%	99.79%	27.02%	99.92%	34.04%	100.00%	100.00%	99.89%
CRS (Top 10 percentile) + VCA-/EBNA1-IgA	CRS >=4.30 and P >=0.98	29.93%	99.79%	18.72%	99.89%	21.11%	100.00%	100.00%	99.87%

Abbreviations: CRS, comprehensive risk score; AUC, area under the curve; PPV: positive predictive value; NPV: negative predictive value.

Supplementary Table 6. The distribution of three EBV variants in samples from southern China and Indonesia.

Southern China	NPC Cases (465)		Controls (589)	
	Low-risk Allele	High-risk Allele	Low-risk Allele	High-risk Allele
EBV162215_C	47 (5%)	845 (95%)	399 (30%)	941 (70%)
EBV162476_C	85 (10%)	807 (90%)	603 (45%)	737 (55%)
EBV163364_T	174 (20%)	718 (80%)	830 (62%)	510 (38%)
**Indonesia**	**NPC Cases (20)**		**Controls (4)**	
	Low-risk Allele	High-risk Allele	Low-risk Allele	High-risk Allele
EBV162215_C	1 (5%)	19 (95%)	0 (0%)	4 (100%)
EBV162476_C	6 (30%)	14 (70%)	0 (0%)	4 (100%)
EBV163364_T	18 (90%)	2 (10%)	4 (100%)	0 (0%)

Supplementary Table 7. Tumor stages of the NPC cases in the training and validation datasets.

	Training dataset cases(n=465)		Validation dataset cases(n=427)	
Tumor Stages*	no.	%	no.	%
I	9	1.94%	7	1.64%
II	50	10.75%	22	5.15%
III	172	36.99%	160	37.47%
IV	210	45.16%	190	44.50%
Missing	24	5.16%	48	11.24%

*Seventh edition (2010) of the AJCC/UICC staging system for NPC.

Supplementary Table 8. EBV variants detected in 19 healthy donors through a three-time re-sampling during a four-week interval.

Tests #	Detection rate*(No.)	Total detectable rate (No.)
Week 0	68% (13)	68% (13)
Week 2	63% (12)	89% (17)
Week 4	74% (14)	95% (18)

* Detection rate calculated by No./Total (19).

Source data are provided in the Source Data file.

Supplementary Table 9. Summary statistics on the seven human SNPs and three EBV SNPs from published studies.

SNP	Gene loci	Chr	Cases	Controls	OR	*P value*	Article
rs2860580_C	*HLA-A*	6	5090	4957	1.72	4.88E-67	Bei JX,Nat Genet. 2010
rs2894207_T	*HLA-B/C*	6	5090	4957	1.64	3.42E-33	Bei JX,Nat Genet. 2010
rs1412829_T	*CDKN2A/2B*	9	6868	9119	1.25	2.80E-08	Bei JX,Cancer Epidemiol Biomarkers Prev. 2016
rs9510787_G	*TNFRSF19*	13	6868	9119	1.16	5.00E-10	Bei JX,Cancer Epidemiol Biomarkers Prev. 2016
rs28421666_A	*HLA-DQ/DR*	6	5090	4957	1.49	2.49E-18	Bei JX,Nat Genet. 2010
rs31489_C	*CLPTM1L/TERT*	5	6868	9119	1.23	6.30E-13	Bei JX,Cancer Epidemiol Biomarkers Prev. 2016
rs6774494_A	*MECOM*	3	6868	9119	1.19	1.50E-12	Bei JX,Cancer Epidemiol Biomarkers Prev. 2016
EBV162215_C	*BALF2/V700L*	EBV	639	652	7.62	1.42E-18	Xu M,Nat Genet. 2019
EBV162476_C	*BALF2/I613V*	EBV	639	652	8.79	9.69E-25	Xu M,Nat Genet. 2019
EBV163364_T	*BALF2/V317M*	EBV	639	652	6.52	2.40E-32	Xu M,Nat Genet. 2019

Abbreviations: Chr, chromosome; OR, odds ratio.

Supplementary Table 10. PCR primers and Unique Extend Primer (UEP) for MassArray genotyping of the seven human SNPs and three EBV SNPs.

SNP_ID	2nd-PCRP	1st-PCRP	UEP-SEQ
EBV162215	ACGTTGGATGACAGCATCAGCACCTTGGAC	ACGTTGGATGACCTGCGACCTGCCAGACCT	CAGCCGCCGGCCGTACA
EBV162476	ACGTTGGATGGTGAGCGGTAAAACAACTGG	ACGTTGGATGTACCACGTGATGCAGTACTC	CGCACGCCGCCTGCCCC
EBV163364	ACGTTGGATGAGGCTGGCATTATATCGGTG	ACGTTGGATGCCTGTTTGCCGACTGTGAG	TATCGGTGTAACGCAGCCA
rs2860580	ACGTTGGATGTGGCAGAAGTGGAAGCAAAC	ACGTTGGATGGGCTTTTCCCTGCTTCATTG	GAAGCAAACCCGTCCTTCTTCA
rs2894207	ACGTTGGATGGCTTATGGTTTCTTCTAAGAG	ACGTTGGATGTGCAAAAGAATAAAGCTGG	GTTTCTTCTAAGAGTTCTCTAAT
rs1412829	ACGTTGGATGCATGCTTTGGGAAACTCTAC	ACGTTGGATGCCATTGCTATGGTTACTATC	CTACCCATGAGATTCATATTCAAGC
rs9510787	ACGTTGGATGGGCTGACCTGCAACTCTTAG	ACGTTGGATGGATTTATTACTTATTGGTGC	gTCATAGTCTTAGAAGACAGC
rs28421666	ACGTTGGATGGTGGTGATGTTTTTATAGCC	ACGTTGGATGCTGAGTGTCATTAAGATCCT	ATCTATACTGTGATATTTATATTTAT
rs31489	ACGTTGGATGTACACTTTCAGCCTGGTGAC	ACGTTGGATGCTCGCATTCCACCTGTTTAC	ACAGCGAGACCTgtTCTCAAAAAAGA
rs6774494	ACGTTGGATGTACGGTAGATGCCATTAAGG	ACGTTGGATGCTATCTTACTTACATTTACC	gcGGAAAACAGTCAATATGTCAC
